# *Plasmodium falciparum *liver stage antigen-1 is cross-linked by tissue transglutaminase

**DOI:** 10.1186/1475-2875-10-14

**Published:** 2011-01-21

**Authors:** William S Nicoll, John B Sacci, Carlo Rodolfo, Giuseppina Di Giacomo, Mauro Piacentini, Zoe JM Holland, Christian Doerig, Michael R Hollingdale, David E Lanar

**Affiliations:** 1 U.S. Military Malaria Vaccine Program, Division of Malaria Vaccine Development, Walter Reed Army Institute of Research, 503 Robert Grant Ave. Silver Spring, MD 20910-7500, USA; 2 Department of Microbiology and Immunology, University of Maryland School of Medicine, Baltimore, MD 21201, USA; 3 Department of Biology, University of Rome Tor Vergata, Rome, Italy; 4 INSERM U609, Wellcome Centre for Molecular Parasitology, University of Glasgow, Glasgow, UK; 5 Consultant to the USMMVP, Malaria Department, NMRC, Silver Spring, MD 20910, USA

## Abstract

**Background:**

*Plasmodium falciparum *sporozoites injected by mosquitoes into the blood rapidly enter liver hepatocytes and undergo pre-erythrocytic developmental schizogony forming tens of thousands of merozoites per hepatocyte. Shortly after hepatocyte invasion, the parasite starts to produce Liver Stage Antigen-1 (LSA-1), which accumulates within the parasitophorous vacuole surrounding the mass of developing merozoites. The LSA-1 protein has been described as a flocculent mass, but its role in parasite development has not been determined.

**Methods:**

Recombinant N-terminal, C-terminal or a construct containing both the N- and C- terminal regions flanking two 17 amino acid residue central repeat sequences (LSA-NRC) were subjected to in vitro modification by tissue transglutaminase-2 (TG2) to determine if cross-linking occurred. In addition, tissue sections of *P. falciparum*-infected human hepatocytes were probed with monoclonal antibodies to the isopeptide ε-(γ-glutamyl)lysine cross-bridge formed by TG2 enzymatic activity to determine if these antibodies co-localized with antibodies to LSA-1 in the growing liver schizonts.

**Results:**

This study identified a substrate motif for (TG2) and a putative casein kinase 2 phosphorylation site within the central repeat region of LSA-1. The function of TG2 is the post-translational modification of proteins by the formation of a unique isopeptide ε-(γ-glutamyl)lysine cross-bridge between glutamine and lysine residues. When recombinant LSA-1 protein was crosslinked *in vitro *by purified TG2 in a calcium dependent reaction, a flocculent mass of protein was formed that was highly resistant to degradation. The cross-linking was not detectably affected by phosphorylation with plasmodial CK2 *in vitro*. Monoclonal antibodies specific to the very unique TG2 catalyzed ε- lysine cross-bridge co-localized with antibodies to LSA-1 in infected human hepatocytes providing visual evidence that LSA-1 was cross-linked *in vivo*.

**Conclusions:**

While the role of LSA-1 is still unknown these results suggest that it becomes highly cross-linked which may aid in the protection of the parasite as it develops.

## Background

The liver stage antigen-1 (LSA-1) is one of the few antigens known to be specifically expressed during the pre-erythrocytic liver stage of *Plasmodium falciparum*[1]. Studies of human immunity following exposure to radiation-attenuated sporozoites, as well as exposure to naturally transmitted parasites, have consistently associated protection with a specific LSA-1 immune response, making LSA-1 an attractive vaccine candidate [2-8]. LSA-1 has undergone several clinical trials. Firstly the sequence of the non-repeat regions were as part of a recombinant pox virus expressing LSA-1 and six other candidate malaria vaccine antigens[9] that induced LSA-1 cellular immune responses[10]. Later it was included as one of five antigens encoded by DNA plasmids that induced boostable cellular responses[11]. Most recently, as a recombinant protein combined with AS01 or AS02 adjuvant[12] it induced high titer antibody and CD4 + T cells that secreted IL-2 and interferon-gamma although it did not induce protection against an experimental *P. falciparum *sporozoite challenge model in humans[13].

Although LSA-1 was first identified in 1987 [14], elucidation of the functional role of LSA-1 has yet to occur. *Plasmodium falciparum *liver-stage parasites are difficult to study, as the only primate model uses chimpanzees [15] and, *in vivo *and liver stages develop in only a few infected hepatocytes. Full liver-stage development of *P. falciparum *occurs *in vitro *in primary hepatocyte cultures from *Aotus *and *Saimiri *monkeys [16] and a human hepatocyte cell line has recently been developed that allows *P. falciparum *infection and development, but again infectivity is extremely low and obtaining protein has thus far proven impossible [17]. This paucity of infected cells, combined with the difficulty of their isolation, results in an inability to biochemically study native liver-stage material.

LSA-1 is a 230 kDa protein characterized by a central repeat region containing 86 repeats of the 17-amino-acid sequence EQQSDLEQERLAKEKLQ or minor variations thereof [18]. Flanking these repeats are a non-repetitive 154 residue N- terminal region and a 280 residue C-terminal region [18,19]. The sequence of LSA-1 repeat and non-repeat regions is highly conserved across strains of *P. falciparum *[19] suggesting a crucial role during liver schizogony [19]. Of interest is the finding that a peptide form the LSA-1 N-terminal region binds to hepatic cells and to HLA-DRβ1*1101[20], which is consistent with the induction of CD4 + T cell responses in clinical trials[11,21]. Analysis of infected primate liver sections probed with antibodies against LSA-1 has shown that synthesis of LSA-1 begins soon after sporozoite invasion and that the protein accumulates throughout the liver stage development [22,23]. From three days post infection, LSA-1 is detectable in the parasitophorous vacuole (PV), which is delineated by the inner plasmalemma and the outer parasitophorous vacuole membrane (PVM) of the infected hepatocyte, and surrounds the developing merozoites as part of a "flocculent mass" [23]. A similar flocculent mass has been observed in *Plasmodium berghei *and *Plasmodium vivax *liver stages [22,24-26], but are not recognized by LSA-1 antibodies. At a later stage, LSA-1 appears to infiltrate the spaces between the pseudocytomeres of the developing schizonts as the plasmalemma forms deep invaginations into the parasite cytoplasm [22,23]. Eventually LSA-1 is localized around the cytomeres just before individualization of the merozoites. Upon hepatocyte rupture the merozoites are released within the flocculent mass into the liver sinusoid where erythrocyte invasion occurs [27-30].

**Figure 1:**
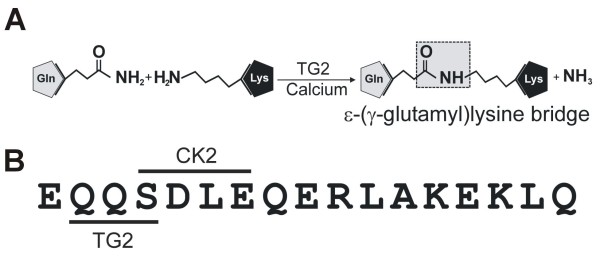
**TG2 cross-linking reaction**. A. In the presence of calcium, TG2 forms an isopeptide bond between γ-carbonyl group of a glutamine residue and the ε-amino group of a lysine residue. Major repeat of LSA-1. B. TG2 and CK2 substrate motifs are indicated by lines.

These observations suggest that LSA-1 is not a soluble protein but has some sort of biochemically-induced structure. LSA-1 central repeat amino acid sequences contain multiple copies of the tripeptide EQQ that is a common substrate for transglutaminases. Transglutaminases, enzymes found in mammals but not protozoa, form ε-(γ-glutamyl)lysine bridges between the acyl donor side chain of glutamine and acyl acceptor side chain of lysine, covalently cross-linking proteins as shown in Figure 1A. Tissue transglutaminase type II (TG2) is a multi-functional enzyme which has been implicated in a range of biological processes including cell death, extracellular matrix stabilization and cell signaling [31-34]. Amongst a range of diseases, TG2 has been implicated as having a role in degenerative conditions of the liver such as hepatitis and Budd-Chiari syndrome [35-39]. Nardacci *et al *[37] demonstrated that a TG2 knockout mouse exhibited impaired liver regeneration after injury and that TG2 is rapidly up-regulated after hepatitis-induced liver damage in human patients.

As purified native protein was not available, recombinant LSA-1 using LSA-NRC that contains the N- and C terminal regions and two repeats[12] was tested as a substrate for TG2 *in vitro*. The data presented here suggests that native *P. falciparum *LSA-1 exhibits TG2-mediated cross-linking *in vivo *that was confirmed by demonstrating that LSA-1 is similarly cross-linked using an *in vivo *mouse-human chimeric model in which *P. falciparum *sporozoites develop into liver stages [40,41]. A physiological role for this cross-linking is proposed.

## Methods

### Production of recombinant LSA N-terminal (LSA-NRC-N) and C-terminal (LSA-NRC-C) proteins

Using the recombinant LSA-1 vaccine candidate construct LSA-NRC [12] as a template, the N- and C- terminal regions were amplified through PCR using the primers GTGGATCCATGGGTACCAACAGCG (N-term fwd); GCGGCCGCAGCAGCTTTTTCTTC (N-term rev); GTGGATCCCGCAAGGCTGACAC (C-term fwd); GCGGCCGCAAGCTTCATAAGTATTTAG (C-term rev). The PCR products were cloned into the pETK expression vector using the compatible BamHI/NotI restriction sites (underlined) [12]. Expression and purification was performed as described for LSA-NRC [12].

### Recombinant TG2 assay (PAGE analysis)

A 500 μl reaction mixture containing 100 mM Tris-HCl pH 6.0; 500 mM NaCl; 10 mM CaCl_2_; 1 mM DTT; 150 μg/ml target protein (LSA-NRC full length, LSA-NRC N-term or LSA-NRC C-term) and 100 μg/ml TG2, was incubated at 37°C for up to 2 h. Small samples (50 μl) were taken at appropriate timepoints. Reaction samples were stopped by the addition of 50 μl of 4× SDS loading dye and stored at 4°C. 25 μl protein samples were separated on precast 4-12% gradient SDS PAGE (Invitrogen, Carlsbad, CA) and stained with Coomassie blue.

### TG2 assay (ELISA Analysis)

An assay based upon Lilley et al.[42] was developed to test LSA-1 cross-linking in vitro. LSA-NRC was bound to 96 well plates at a concentration of 0.20 μg/ml in 50 mM Na_2_CO_3 _at pH 9.8 for 1 h at 37°C. Wells were then blocked for 1 h at 37°C with 200 μl of a solution containing 0.5% boiled casein; 1% Tween 80; 50 mM Na_2_CO_3 _pH 9.8. Wells were washed twice with 1×PBS pH 7.4; 0.05% Tween 80 and twice with H_2_O. Reactions were set up with 100 mM Tris-HCl pH 6.0; 5 mM CaCl_2_; 10 mM DTT; 5 μg/ml biotinylated LSA-NRC (produced using the EZ-link NHS-biotin labeling kit, Pierce, Rockford, IL); and up to 2 μg/ml TG2 in a total reaction volume of 50 μl. Reactions were incubated for 1 h at 37°C. Wells were washed twice with 1×PBS pH 7.4; 0.05% Tween 80 and twice with H_2_O. Plates were then incubated with 50 μl of 1:10,000 dilution of peroxidase-bound neutravidin; 100 mM Tris-HCl, pH 8.5; 0.5% boiled casein for 1 h at room temp. Wells were washed three times with 1×PBS, pH 7.4; 0.05% Tween 80 and twice with H_2_O. Developing was performed using 100 μl of KPL ABTS peroxidase substrate (KPL Inc., Gaithersburg MD) for 60 min. Development was stopped with 100 μl 1% SDS and samples were read at 405 nm.

### Recombinant TG2 cell extract assay (Western analysis)

To assess whether LSA could act as a TG2 substrate an *in vitro *assay was developed with cell extracts from both human neuroblastoma cell lines either not expressing (SK-N-BE-2), or over-expressing (TGA), hTG2 [43]. For the cell free assay, 500 ng of LSA protein were incubated with 250 μg of cell extracts in 50 mM Tris-HCl, pH 8.3, 30 mM NaCl, 10 mM DTT, 15 mM CaCl_2 _at 37°C, in a final volume of 50 μl. Every 5 min 10 μl of the reaction were taken and after addition of 2 mM EGTA and NuPAGE sample buffer, samples were boiled and separated on 4-12% NuPAGE gel (Invitrogen) prior to analysis by Western blot. Blots were probed with 1:1,000 dilution of anti-LSA polyclonal mouse primary antibody and 1:1,000 dilution of an HRP-conjugated goat anti-mouse secondary antibody.

### Reversed-phase HPLC analysis

RP-HPLC was performed using a Waters modular HPLC system consisting of two Waters 510 fluid pumps, Waters 717plus autoinjector, Waters 2487 UV detector, Waters system interface module and a Waters DeltaPak C18-300A column. Instrument control, data acquisition and evaluation were performed using Waters Millenium32 software. Buffer A comprised 0.05% (v/v) trifluoracetic acid in H_2_O, whereas buffer B comprised 0.05% (v/v) trifluoroacetic acid in acetonitrile. Bound proteins were eluted using a linear gradient of 10-100% buffer B over 30 min at a flow rate of 1 ml/min. Peaks were analysed by matrix-assisted laser desorption ionization-time of flight mass spectrometry (Voyager biospectrometry RP system; Applied Biosystems) with a α-cyano-4-hydroxycinnamic acid (peptides) or sinapinic acid (protein) matrix.

### Mouse/human chimeric liver immunofluorescence assays

All experiments utilized sporozoites of the NF54 strain of *P. falciparum*. Sporozoites were reared in *Anopheles stephensi *mosquitoes and were isolated by hand dissection or by a discontinuous Renografin gradient [44] in Medium 199 (Gibco, Grand Island, NY) with 5% foetal calf serum. The generation of chimeric mice has been previously described [40,41]. Briefly, SCID mice, homozygous for the urokinase type plasminogen activator transgene (SCID *Alb-uPA*), were inoculated intrasplenically with 1 × 10^6 ^human hepatocytes. At 6 wks post-transplant, serum analysis for human alpha one antitrypsin (hAAT) by ELISA was performed to determine the success of the transplantation. Mice that demonstrated >25 μg/ml hAAT were then used for infection with *P. falciparum *sporozoites. Mice were cared for by the University of Alberta Health Sciences Laboratory Animal Services according to the guidelines of the Canadian Council on Animal Care and under protocols approved by the University of Alberta Faculty of Medicine and Dentistry Health Sciences Laboratory Animal Ethics Committee. Additionally, the experiments reported here were carried out according to the principles set forth in the "Guide for the Care and Use of Laboratory Animals"[45]

### Infection with sporozoites and tissue collection

Chimeric mice received an intravenous tail vein injection of 1-1.5 × 10^6 ^*P. falciparum *sporozoites and were subsequently euthanized by CO_2 _overdose at several different timepoints post-infection and their livers removed for cryosectioning. Livers were rinsed in PBS, the lobes cut into separate pieces and frozen in Tissue-Tek O.C.T. compound (Miles Scientific, Naperville, IL.) using an isopentane/liquid N_2 _bath. Tissue cryo-sections (7 μm) were then cut, fixed in absolute methanol, and stored at -80°C until used.

### Immunofluorescence assay

Slides were removed from the freezer, placed in a desiccator and allowed to equilibrate to room temperature. The diluted antiserum (polyclonal rabbit anti-LSA-1 [12] or 71A3F1 and 81D1C2 monoclonal (Abcam Inc, Cambridge, MA) antibodies) was then applied to the tissue section in a volume sufficient to cover the tissue. Slides were incubated for 30 min at 37°C in a humidity chamber, then washed three times for 5 min with PBS and incubated with a fluorescein conjugated IgG (Kirkegaard and Perry, Gaithersburg, MD) diluted 1:40 with 0.02% Evan's blue for 30 min at 37°C. The Evan's blue was added to act as a counterstain to suppress any autofluorescence in the tissue. The specificity of the secondary antibody varied depending upon the species of the primary antibody used to stain the sections. Sections were then washed as above and the slides mounted with Vectashield^® ^mounting media (Vector Labs, Burlingame, CA). The stained slides were screened with a Nikon Eclipse E600 epifluorescent microscope and digital images collected with a SPOT digital camera (Diagnostic Instruments, Inc., Sterling Hgts, MI).

### CK2 phosphorylation assay

Recombinant *P. falciparum *CK2α (PfCK2 α) (PlasmoDB ID PF11_0096) was cloned and expressed in *E. coli *as a GST-tagged protein (Z. Holland and C. Doerig, unpublished data). Kinase assays were performed in a standard reaction (30 μl) containing 15 mM Tris-HCl, pH 7.5; 15 mM MgCl_2_; 1.5 mM MnCl_2_; 10 mM β-glycerol phosphate; 10 mM NaF; 10 μM ATP; 0.075 MBq of [g-^32^P]ATP (220 TBq/mmol; GEHealthcare), 6 μg of substrate (LSA-NRC) and 1 μg of recombinant PfCK2α. After 30 min at 30°C, reactions were terminated by the addition of Laemmli buffer, boiled for 3 min, and separated on a 12% SDS/polyacrylamide gel. Following staining with Coomassie blue, the gel was dried and submitted to autoradiography.

## Results

### LSA-1 contains substrate motifs for TG2 and casein kinase II

BlastN and BlastP searches against the full-length *P. falciparum *LSA-1, the N-terminal, repeat and C-terminal regions have failed to reveal the existence of homologous genes in any other organism, including all other known *Plasmodium *species, except *Plasmodium reichenowi*. Motif searches of the LSA-1 amino acid sequence revealed that the 17-mer repeat region possesses the properties of a glutamine acyl-donor substrate for TG2 as well as an immediately adjacent casein kinase II (CK2) substrate motif (Figure 1B). Substrates of TG2 are wide and varied, as is the TG2 substrate motif, however, it is generally considered that proteins containing two or more adjacent glutamines are good TG2 substrates [46,47]. Additionally, for the lysine substrate, increased specificity is seen when the residue on the N-terminal side of the lysine is a hydrophobic amino acid such as leucine [48].

### LSA-1 is a substrate for TG2

**Figure 2:**
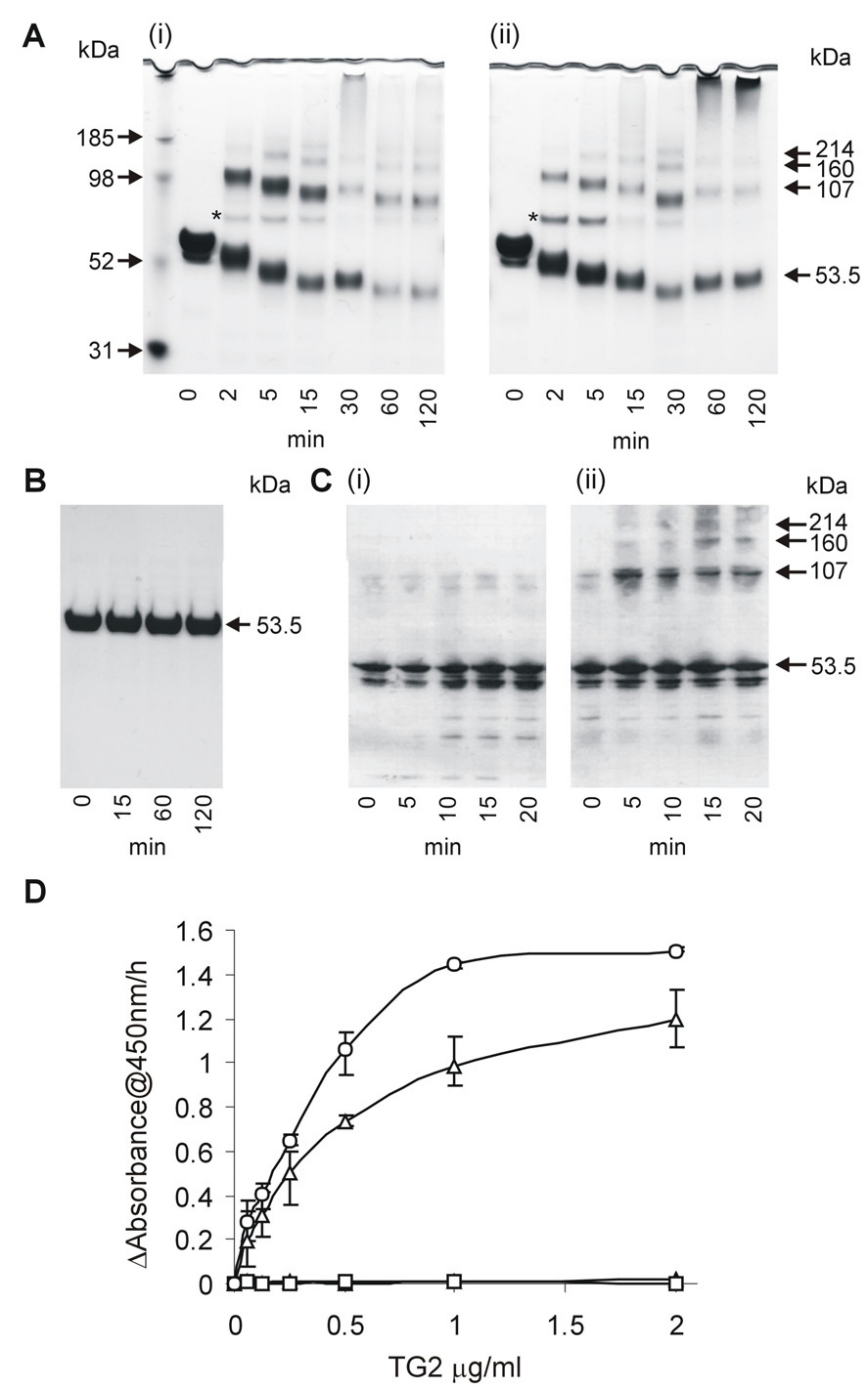
**Assessment of LSA-NRC cross-linking by TG2**. A. SDS PAGE analysis of LSA-NRC samples after various times of incubation with 100 μg/ml of either gpTG2 (i) or hTG2 (ii). * indicates the band representing TG2 (MW - 76.6 kDa). B. SDS PAGE analysis of LSA-NRC samples after various times of incubation with 100 μg/ml of gpTG2 in the absence of CaCl_2 _indicating dependence of cross-linking on Ca^+^. C. Western analysis of LSA-NRC samples after incubation with lysates of human cell line SK-N-BE(2) (i) or its stably transfected derivative, TGA, that over-expresses hTG2 (ii). Blots were probed with anti-LSA-NRC polyclonal antibodies. D. Plate based colorimetric analysis of LSA-NRC TG2 mediated cross-linking. Change in absorbance at 405 nm is shown as a function of TG2 concentration. Open circles - hTG2; Open triangles - gpTG2; Open squares - gpTG2 in the absence of CaCl_2_; closed triangles - in the absence of TG2. Error bars show variation of 3 experiments.

The recombinant form of LSA-1 (LSA-NRC) contains the N- and C- terminal regions combined with two of the central repeats [12]. To assess whether this recombinant LSA-NRC could act as a TG2 substrate, LSA-NRC was incubated in the presence of 50 μg/ml guinea pig liver TG2 (gpTG2). gpTG2 was chosen as it is the most widely available TG2, is widely used in TG2 assays, and is known to have a wide substrate range [49]. Additionally, since LSA-1 is more likely to come into contact with human TG2 (hTG2), LSA-NRC was assessed whether it could act as a substrate for both purified recombinant hTG2, and hTG2 in cell lysates from a transgenic human cell line that over expresses hTG2. Figure 2A (i and ii) clearly shows the production of LSA-NRC multimers over time after incubation with gpTG2 or hTG2. As time progressed a flocculent precipitate was observed in the reaction tube which was unable to enter the PAGE gel, as can be seen in the tops of the wells in the late time points of Figure 2A(i and ii). The largest molecule that can be seen on the gel is a small amount of a 214 kDa protein, which would fit the size of an LSA-NRC tetramer. It is assumed that multimers bigger than this precipitate out of solution.

An increase in mobility can be seen over time for the TG2-treated LSA-NRC monomer (Figure 2A i and 2A ii). Incubation of LSA-NRC with TG2 (Figure 2B) in the absence of CaCl_2 _did not result in any detectable cross-linking.

Figure 2C shows a Western blot analysis of LSA-NRC incubated with lysates of human cell line SK-N-BE(2) (i) and its stably transfected derivative, TGA, that overexpresses hTG2 (ii). Although a small amount of a band that correlates to LSA-NRC dimers can be seen at time zero in both SK-N-BE(2) and TGA lysate-treated LSA-NRC, no further cross-linking is seen in cell lysates not expressing hTG2, whereas in contrast several bands attributed to LSA-NRC cross-linking are observed in lysates containing hTG2.

To further quantify TG2 activity, an ELISA assay was developed (based on [42]). As can be see in Figure 2D an increasing concentration of TG2 is directly related to an increasing level of cross-linked biotin-labeled LSA-NRC. Figure 2D clearly illustrates that no cross-linking occurs in the absence of either calcium or TG2, confirming that this reaction is not autocatalytic and is calcium dependent as is typical of TG2 reactions.

### The LSA-1 repeat region is the target Of TG2 cross-linking

**Figure 3:**
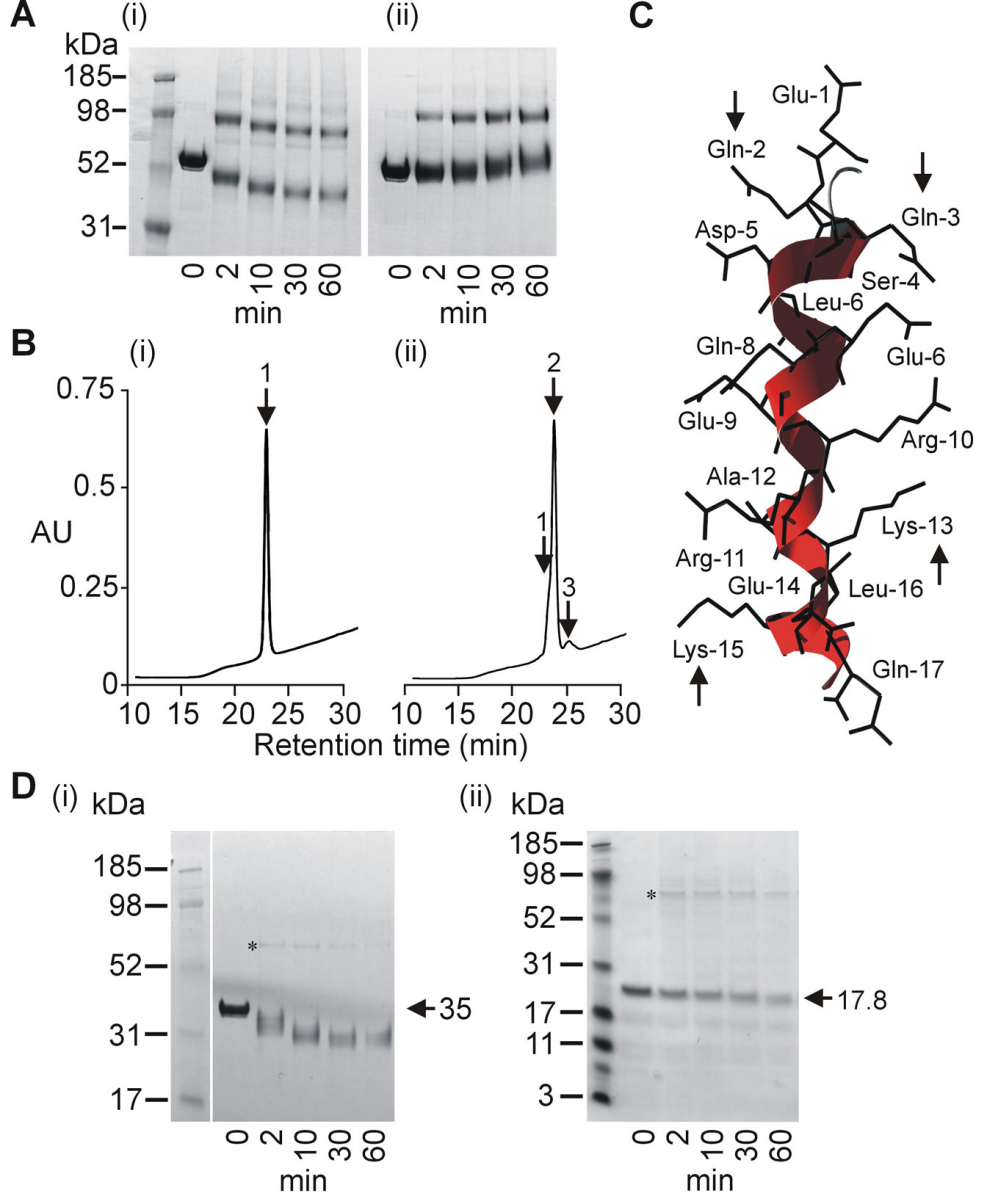
**Analysis of cross-linking site**. A. PAGE analysis of LSA-NRC TG2-cross-linking in the absence (i) or presence (ii) of peptide corresponding to the major repeat sequence of LSA-1. B. RP-HPLC analysis of a peptide corresponding to the major repeat sequence of LSA-1 before (i) and after (ii) gpTG2 treatment for 2 h at 100 μg/ml gpTG2. Position of monomers [retention time 23.3 min] (1), dimers [retention time 24.5 min] (2) and trimers [retention time 25.6 min] (3) are indicated. (ii). C. Tertiary structure of a single LSA-1 major repeat as predicted by Robetta modeling. Arrows indicate glutamines and lysines predicted to be involved in TG2 mediated cross-linking. D. PAGE analysis of gpTG2 cross-linking of LSA-NRC-C (i) and LSA-NRC-N (ii). * indicates band formed by the gpTG2 enzyme (MW - 76.6 kDa).

To assess whether the predicted TG2 glutamine substrate in the LSA-1 repeat region was in fact a TG2 substrate, LSA-NRC was incubated with TG2 and a LSA-1 repeat peptide containing a single repeat unit. As can be seen in Figure 3A, inclusion of the peptide resulted in blocking the shift in mobility, suggesting a reduction in intra-LSA-NRC cross-linking. Interestingly, the mobility of LSA-NRC-peptide decreased over time suggesting that multiple repeat peptides are being successively crosslinked to the LSA-NRC monomer, gradually increasing its molecular weight.

To assess the ability of the LSA-1 repeat region to crosslink to itself, a single repeat peptide was incubated with gpTG2. RP-HPLC analysis of the cross-linking reaction showed three distinct peaks (Figure 3B). Analysis of the peaks by MALDI-TOF MS showed that peaks 1, 2 and 3 related to the expected sizes of monomers, dimers and trimers of the LSA repeat peptide (data not shown). Analysis of the primary amino acid sequence of the LSA-1 repeat peptide by Robetta Protein Structure Prediction server[50] yielded the tertiary structure shown in Figure 3C. Of note are the lysine glutamine pairs (Gln-2/Lys-15 and Gln-3/Lys-13) that project out on either side of the helix that could act as anti-parallel TG2 cross-linking pairs, and thus allow the formation of multimers.

To further assess the role of TG2 cross-linking of the LSA-1 repeats, recombinant versions of both the N-terminal (LSA-NRC-N), and C-terminal region of LSA-NRC (LSA-NRC-C) were produced that contained none of the central repeats. Incubation of LSA-NRC-N and LSA-NRC-C with gpTG2 did not result in multimers being produced (Figure 3D i and 3D ii). However, a similar increase in mobility was seen for the monomers of LSA-NRC-C as was seen for monomers of LSA-NRC.

### CK2 phosphorylation does not affect TG2 cross-linking

**Figure 4:**
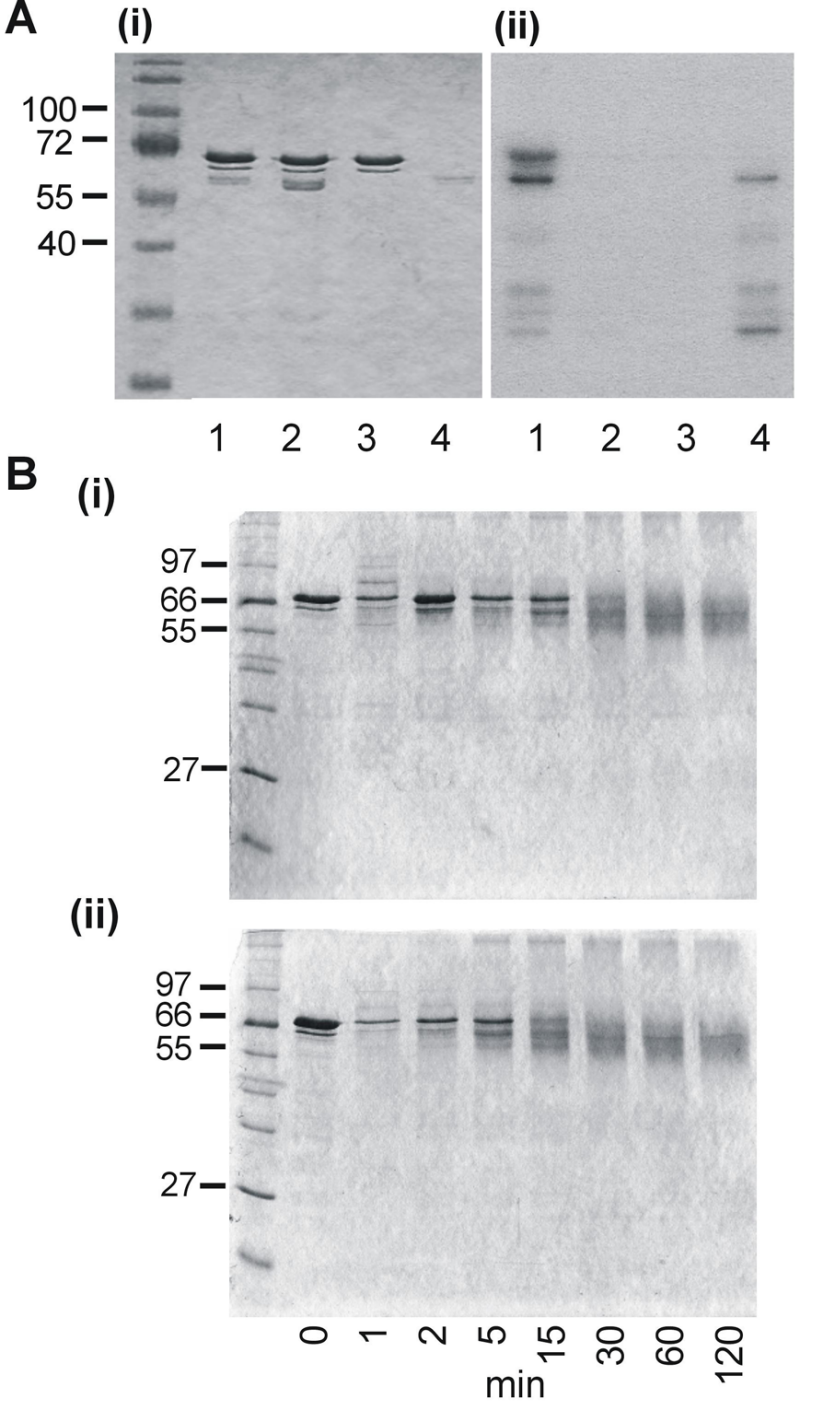
**PfCK2α regulation of TG2 LSA-1 cross-linking**. (A) PAGE analysis of LSA-NRC incubated with PfCK2 α. Coomassie stained samples (i). Autoradiograph of gel in (i) (ii). Lane 1 - LSA-NRC incubated with PfCK2α; lane 2 - LSA-NRC incubated with inactivated PfCK2α; lane 3 - LSA-NRC; lane 4 - PfCK2α. (B) PAGE analysis of samples taken at various time points from non-phosphorylated (i) and phosphorylated (ii) LSA-NRC incubated with gpTG2.

The presence of multiple CK2 phosphorylation sites (one per repeat) in the repeat region of LSA-1 suggests the possibility of TG2 mediated cross-linking being regulated through casein kinase 2 (CK2) phosphorylation. To test this hypothesis, a recombinant catalytic subunit of *P. falciparum *CK2, PfCK2α was prepared. Initially, to ascertain whether LSA-1 can be phosphorylated by CK2, LSA-NRC was incubated with [g-^32^P]ATP in the presence or absence of PfCK2α. As can be seen on the Coomassie blue stained-gel Figure 4A(i), LSA-NRC is present in lanes 1-3, but only the lane containing LSA-NRC and active PfCK2α shows a band on the autoradiograph indicating incorporation of ^32^P into the LSA-NRC sample (Figure 4A(ii) lane 1). No bands of this size can be seen in any of the control lanes, which include a reaction with a kinase-mutant (K72M) of the enzyme (Figure 4A (ii) lanes 2-4). To assess the effect of phosphorylation on TG2 mediated LSA-NRC cross-linking, phosphorylated and non-phosphorylated LSA-NRC were treated with gpTG2. As can be seen in Figure 4B(i) and (ii), phosphorylation caused no detectable difference to gpTG2 under the conditions used.

### LSA-1 cross-linking in vivo

**Figure 5:**
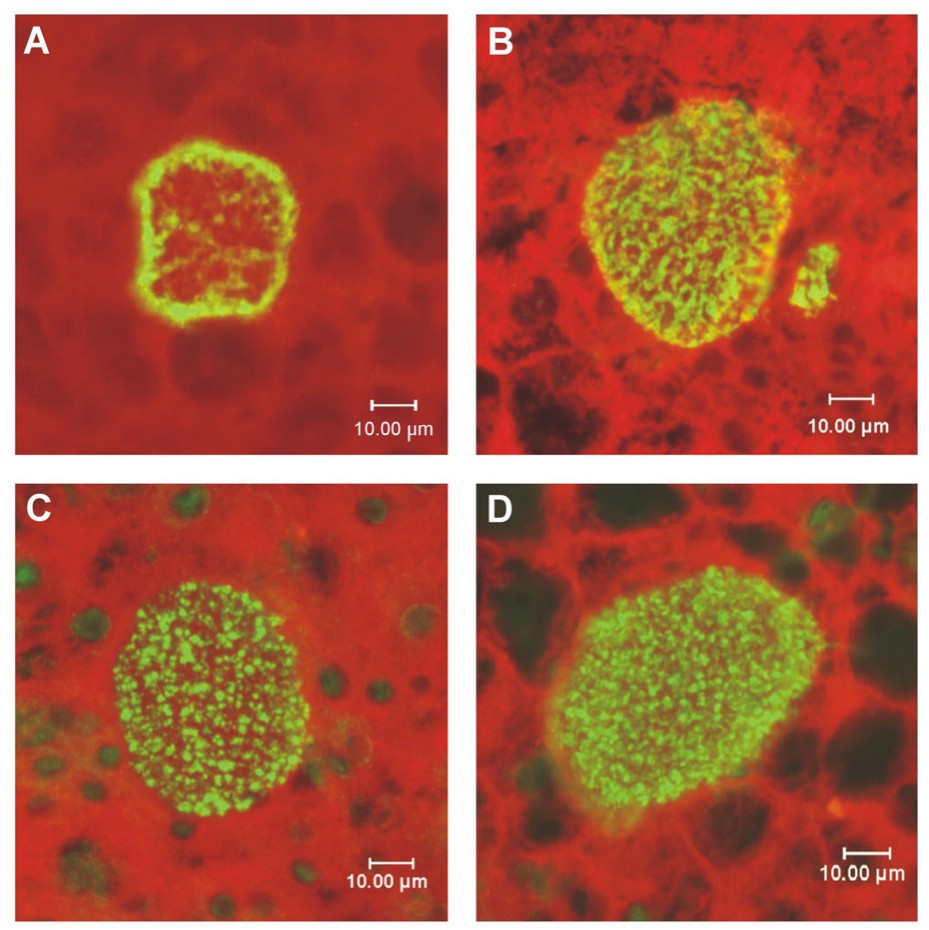
***P. falciparum *LSA-1 in human liver hepatocytes**. *P. falciparum *sporozoites were injected intravenously into transgenic, chimeric mice possessing functioning human livers. Liver nodules were collected 5 or 6 days after injection, fixed and sectioned. Sections containing developing parasites were probed with antibody and detected by immunofluorescence. (A) A 5-day infected liver section probed with mouse polyclonal sera against LSA-NRC. (B) A 6-day infected liver treated as in (A). (C) A 6-day infected liver probed with mAb 71A3F1 that recognizes the TG2 formed isopeptide bond between glutamine and lysine. (D) As in (C) but using another mAb, 81D1C2, that also recognizes the TG2 isopeptide bond [52]

*Plasmodium falciparum *is a human parasite and does not develop in animals except for a few species of non-human primates. Therefore, the isolation of infected hepatocytes from *in vivo *sporozoite infection under normal conditions is virtually impossible. Likewise, the *in vitro *tissue culture of hepatocytes that are susceptible to sporozoite invasion is limited and does not yield sufficient material for biochemical analysis. Fortunately, a chimeric mouse model has recently been developed wherein human livers are grown [41]. Because the pattern of LSA-1 in developing liver schizonts is so distinctive it predicted that monoclonal antibodies specific to the glutamine-lysine isopeptide bridge should demonstrate the same staining pattern as anti-LSA-1 antibodies. Therefore, to assess whether LSA-1 is crosslinked *in vivo*, *P. falciparum *infected liver sections from the chimeric mice were probed with polyclonal mouse antibodies raised against LSA-NRC. LSA-1 is clearly visible in infected hepatocytes at day 5 and day 6 post-infection (Figure 5A and 5B). To detect specific glutamine-lysine isopeptide linkages created by TG2 cross-linking, two different mouse monoclonal antibodies specific for this linkage (71A3F1 and 81D1C2) were used to probe fixed tissue sections (Figure 5C and 5D). Fluorescent signal is seen across the entire infected cell in a similar pattern to that seen with anti-LSA-1 antibodies. In contrast, the surrounding non-infected cells used as a control for non-parasite protein reactivity exhibit almost no fluorescence.

## Discussion

LSA-NRC is susceptible to TG2 cross-linking by both gpTG2 and hTG2 *in vitro*. As a monomer LSA-NRC is highly soluble, but upon cross-linking, LSA-NRC rapidly comes out of solution and is seen as a flocculent mass under *in vitro *cross-linking conditions. This is consistent with ultrastructural observations [19,23] that described LSA-1 in 6 day post-infection primate liver sections as a 'fluffy flocculent mass'.

The presence of a potential CK2 phosphorylation site within the LSA-1 repeat region that overlaps the TG2 cross-linking site suggested that TG2-mediated cross-linking of LSA-1 may be regulated through CK2 phosphorylation. However, although this study demonstrated that LSA-NRC is phosphorylated *in vitro *by CK2 of parasite origin, this phosphorylation does not affect TG2 mediated cross-linking under our experimental conditions. However, it cannot be ruled out that phosphorylation has an effect on cross-linking, but that the proportion of phosphorylated substrate is too small in our conditions to allow detection in the cross-linking assay.

Tertiary structural Robetta modelling [51] predicted that each LSA-1 repeat sequence exists as a single α-helix resulting in an extended α-helical arrangement. This is consistent with previous analysis of the LSA-1 repeat peptides by circular dichroism suggesting that the repeat region of LSA-1 is an uninterrupted stretch of α-helices reaching a length of 220 nm [19]. The α-helix model produced by Robetta modelling in this study showed that a Gln-Lys pair protrudes on either side of the repeat helix. By orientating successive LSA-1 molecules in opposite directions these pairs could bind to each other forming TG2-cross-linked bonds between molecules resulting in a flexible matrix type arrangement as seen with the transglutaminase-mediated cross-linking of fibrin during blood clotting [52]. Incubation of the LSA-1 repeat peptide with gpTG2 resulted primarily in the formation of peptide dimers with very few trimers or tetramers, indicating that the majority of cross-linking was occurring at only one site on the peptide and that once this is bound no further cross-linking occurs. Further evidence indicating that the primary cross-linking site is the repeat region was provided by attempts to crosslink LSA-NRC-N and LSA-NRC-C proteins that lack any repeats: neither of these proteins was able to form multimers after incubation with TG2. However, LSA-NRC-C did show an increase in mobility during SDS-PAGE analysis suggesting that intramolecular cross-linking was occurring and leading to speculation that intramolecular cross-linking of the C-terminal of LSA-NRC may be responsible for the increased mobility seen in the full length LSA-NRC.

Obtaining human or primate livers infected with early stages of *P. falciparum *is either impossible or prohibitively expensive. Therefore, analysis of infected human liver sections derived from chimeric mice infected with *P. falciparum*[40,41] has proved invaluable. That TG2-specific cross-linking does occur *in vivo *and that the location of this cross-linking is closely associated with that of LSA-1 was shown by incubating tissue sections derived from these livers with two different monoclonal antibodies that are specific to the very unique bond formed by the TG2 cross-linking, the ε-(γ-glutamyl)lysine cross-bridge. While this model system does provide tissue sections for analysis, the infection rate is not sufficient to allow purification of native LSA-1, and thus biochemical or biophysical analysis that would show that native LSA-1 is cross-linked by TG2. However, the *in vitro *data coupled with the *in vivo *co-localization of the unique ε-(γ-glutamyl)lysine cross-bridge with the LSA-1 tissue localization pattern observed strongly suggests the two are associated *in vivo*.

This then leads to speculation as to why LSA-1 needs to be cross-linked during infection. The internal repeat unit of LSA-1, about 85 copies of a 17 amino acid unit containing the TG2 substrate motif would suggest that its function is important. A typical *P. falciparum *infection involves the migration of the *P. falciparum *sporozoites through a number of liver cells prior to actually infecting a hepatocyte and forming a parasitophorous vacuole [53]. Cellular damage to the liver has been shown to result in up regulation of TG2 expression in the damaged tissue [54]. Additionally, TG2 activity has been shown to be present in *P. falciparum *and *Plasmodium gallinaceum *infected red blood cells [55]. Thus it is likely that TG2 activity would be found at the site of *P. falciparum *infection. A *P. falciparum *infected hepatocyte experiences major internal reorganization as the parasite schizonts undergo massive expansion, with tens of thousands of merozoites being made in each infected cell. It is reasonable to speculate that in order to maximize the survival rate of the merozoites it would be advantageous for the parasite to maintain structural integrity of the host cell for as long as is feasibly possible. Construction of a dense cytoskeletal matrix formed with crosslinked LSA-1 would be possible to create a strong flexible cell that would allow rapid expansion but minimize the chance of rupture. However, if this were the case, why is LSA-1 protein not found in most other *Plasmodium *species? It is possible that a similar flocculent material seen in other Plasmodium species is functionally analogous to LSA-1, but differs in sequence, and a possible functional ortholog, identified by synteny mapping [56] in *Plasmodium berghei*, that may play a similar role.

It has recently been shown in *P. berghei *that merozoites are released in 'merosomes' - clusters of merozoites that bud off from the main hepatocyte, taking a protective layer of the host membrane with them [29]. Prior to merosome formation, *Plasmodium *liver stages seem to protect the host cell from apoptosis [57] through hepatocyte growth factor (HGF) signaling of its receptor MET, but may undergo autophagy induced by the huge growth of the liver stage parasite [30]. HGF/MET signaling may also occur during sporozoite invasion of hepatocytes, again blocking apoptosis. LSA-1, or analogous flocculent material, may therefore play a vital role in maintaining cell integrity during autophagy and merosome formation, TG2 has been shown to play an essential role in conferring resistance to damage in the liver [37]. *Plasmodium falciparum *may be using this response to maintain the structural integrity of the infected hepatocyte. Reinforcement of the cell by a LSA-1 matrix could play a role in reducing the chance of hepatocyte death by apoptosis.

These studies suggest that recombinant LSA-1 is a TG2 substrate *in vitro *and that the unique modification made by TG2 to the protein can be detected *in vivo *in a pattern consistent with LSA-1 protein localization; this is the first study suggesting a functional role for LSA-1.

## Competing interests

The authors declare that they have no competing interests.

## Authors' contributions

WSN, MRH and Del conceived the study. WSN, JBS, GdG, MP and CD designed the experiments. WSN, JBS, CR and ZJMH performed the experiments. WSN and DEL analysed the data. WSN, MRH and DEL wrote the manuscript. DEL and MRH revised the manuscript. All authors read and approved the final manuscript.
